# Biofunctional Constituents from *Michelia compressa var. lanyuensis* with Anti-Melanogenic Properties

**DOI:** 10.3390/molecules200712166

**Published:** 2015-07-03

**Authors:** Chia-Wei Chu, Chi-Ming Liu, Mei-Ing Chung, Chung-Yi Chen

**Affiliations:** 1 School of Pharmacy, College of Pharmacy, Kaohsiung Medical University, Kaohsiung 807, Taiwan; E-Mail: eric.chu@astellas.com; 2 Department of Nursing, Tzu Hui Institute of Technology, Pingtung County 926, Taiwan; E-Mail: beagleliu@gmail.com; 3 Department of Nutrition and Health Science, School of Medical and Health Sciences, Fooyin University, Kaohsiung 831, Taiwan

**Keywords:** *Michelia compressa var. lanyuensis*, Magnoliaceae, tyrosinase activity, melanin

## Abstract

Seven compounds were extracted and purified from the roots of *Michelia compressa* *var. lanyuensis*. These compounds are liriodenine, (−)-*N*-acetylanonaine, pressalanine A, *p*-dihydroxybenzaldehyde, 3,4-dihydroxybenzoic acid, (−)-bornesitol and β-sitostenone. These compounds were screened for anti-proliferation and anti-tyrosinase activities in B16F10 cells. Liriodenine, pressalanine A, (−)-bornesitol and β-sitostenone displayed cytotoxicity at high concentration (100 μM), but liriodenine (5 μM), (−)-*N*-acetylanonaine (10 μM), and β-sitostenone (5 μM) inhibit tyrosinase activity and reduce the melanin content in B16F10 cells without cytotoxicity, suggesting that liriodenine and β-sitostenone could be safe and potentially used in cosmetic skin whitening.

## 1. Introduction

The genius *Michelia* (family Magnoliaceae) has 30 species and is distributed in Asia. Many bioactive compounds have been isolated from the Magnoliaceae, including alkaloids, aporphines, linalool and so on [1,2]. The pharmacological activities of bioactive compounds from the Magnoliaceae include anticancer, anti-inflammatory, and antibacterial effects [2,3,4,5]. *Michelia compressa var. lanyuensis* is an evergreen tree mainly distributed in Taiwan and Japan. In previous studies, the compounds from *Michelia compressa var. formosana* have been reported [2,6,7,8].

Tyrosinase is well-known enzyme in the synthesis of the human pigment melanin responsible for coloring skin, eyes and hair. In animals, the melanin pigments are produced by melanocytes [9,10]. The abnormal accumulation of melanin pigments is responsible for hyperpigmentations, including freckles and nevus. Skin hyperpigmentation is the most common complaint of patients. Depigmenting agents are very important in the cosmetic and medicinal industries. They should provide safety and efficacy without side effects. In the clinic, whitening agents are used for treating dermatological disorders related to melanin hyperaccumulation. UV exposure, sunburn and family history can cause skin cancers [11], which can be divided into non-melanoma and melanoma skin cancers. Non-melanoma skin cancer includes squamous cell carcinoma [12]. Non-melanoma skin cancer is common in Western countries, especially in the United States. Melanoma has a poor prognosis and low patient survival rates.

**Figure 1:**
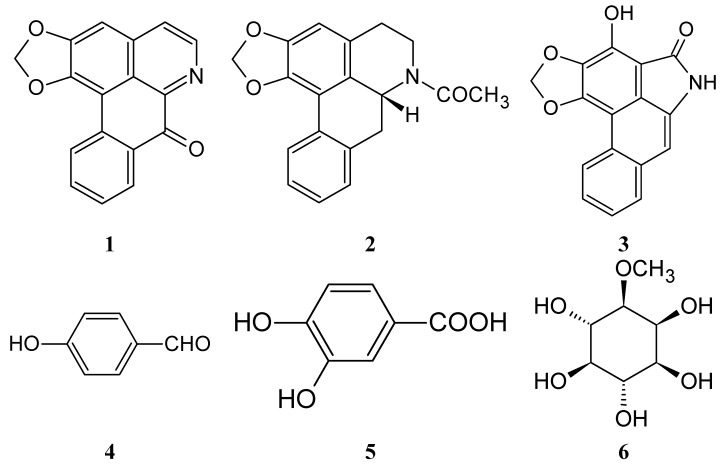

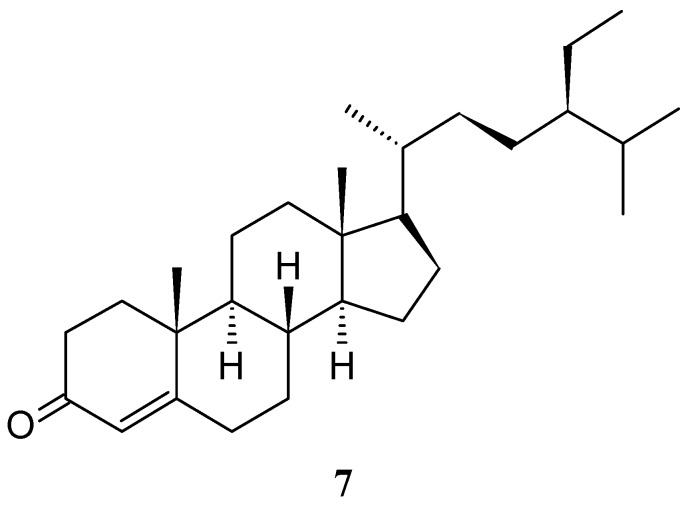
Structures of alkaloids **1**–**7**isolated from the roots of *M.* *compressa var. lanyuensis.*

As part of our ongoing research program to isolate and identify bioactive compounds from natural sources, in this work the bioactive constituents of the roots of *Michelia compressa var. lanyuensis*. were further investigated by assaying their anticancer effects and tyrosinase and cellular proliferation inhibition, We isolated seven bioactive compounds, namely liriodenine (**1**), (−)-*N*-acetylanonaine (**2**), pressalanine A (**3**), *p*-dihydroxybenzaldehyde (**4**), 3,4-dihydroxybenzoic acid (**5**), (−)-bornesitol (**6**) and β-sitostenone (**7**). Pressalanine A (**3**), 3,4-dihydroxybenzoic acid (**5**) and (−)-bornesitol (**6**) were isolated for the first time from this plant. The structures of these seven compounds are summarized in Figure 1.

## 2. Results and Discussion

### 2.1. Anti-Proliferative Properties of Compounds ***1**–**7*** from M. compressa var. lanyuensis on B16F10 Cells

**Figure 2:**
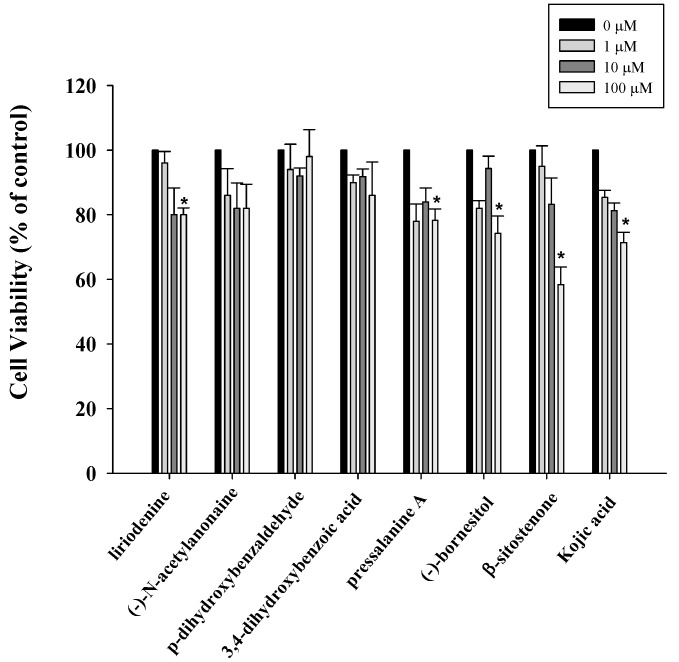
Anti-proliferative effects of compounds on B16F10 cells. Cell growth was determined by XTT assay after incubation with 1, 10, 100 μM of different compounds. Results are expressed as the percent of the cell proliferation of the vehicle control at 24 h. Values are expressed as means ± S.D. of *n* = 3. * *p* < 0.05 *vs.* vehicle control group.

The XTT assay is a colorimetric assay used to measure cell viability. The XTT assay was used to investigate the anti-proliferation activity of tested compounds in B16F10 cells after 24 h treatment. The cells were treated with compounds at different concentrations (1, 10, and 100 μM) as demonstrated in Figure 2. Liriodenine (**1**), pressalanine A (**3**), (−)-bornesitol (**6**) and β-sitostenone (**7**) produced cytotoxicity at high concentration (100 μM). Below 10 μM none of these compounds displayed cytotoxicity in B16F10 cells after 24 h treatment. Kojic acid (positive control) showed dose-dependent inhibition in B16F10 cells, whereby at 10 and 100 μM it slightly inhibited cell growth (Figure 2).

### 2.2. Inhibitory Effects of the Test Compounds on Tyrosinase Activity and Melanin Content 

Melanin is a vitally important factor in determining the skin color of humans. The melanogenesis pathway consists of the enzymatic hydroxylation of l-tyrosine and the oxidation of l-dopa to dopaquinone [13]. The whitening effects of compounds and kojic acid (positive control) were evaluated by measuring melanin content in B16F10 cells. The cellular tyrosinase-inhibition abilities of the compounds from *M. compressa var. lanyuensis* were also examined in B16F10 cells (Table 1). In order to confirm the inhibition of enzyme activity, the effect of test compounds were evaluated by measuring tyrosinase activity with l-tyrosine as substrate. Liriodenine (**1**) and β-sitostenone (**7**) showed minor tyrosinase activity inhibition, with 14.52% ± 3.58% and 25.48% ± 6.30% inhibition on B16F10 cells (5 μM). In contrast, 3,4-dihydroxybenzoic acid (**5**) significantly increased the tyrosinase activity in B16F10 cells at the concentration of 5 μM. Further, we also observed that liriodenine (**1**), (−)-*N*-acetylanonaine (**2**) and β-sitostenone (**7**) showed a small decrease in the melanin content in B16F10 cells. Kojic acid (10 μM) inhibited the tyrosinase activity by 25.38% ± 1.40% and decreased melanin content (20.00% ± 2.12%) in B16F10 cells. We discovered that the decrease in melanin contents matched the tyrosinase activity inhibition with the same tendencies. Based on the XTT, tyrosinase activity and melanin content results, liriodenine (**1**) and β-sitostenone (**7**) might have a potential use in skin lightening without cytotoxicity and safety in skin whitening.

**Table 1:** The inhibitory effects of of *M. compressa var. lanyuensis* isolated compounds on tyrosinase activity and melanine content formation in melanoma cells.

Compound	Inhibition (%) Tyrosinase Activity	Inhibition (%) Melanin Content
Liriodenine (**1**) (5 μM)	14.52 ± 3.58	19.03 ± 4.55
(−)-*N*-Acetylanonaine (**2**) (10 μM)	7.75 ± 1.42	17.70 ± 3.18
Pressalanine A (**3**) (10 μM)	4.84 ± 0.80	−12 ± 4.58
*p*-Dihydroxybenzaldehyde (**4**) (10 μM)	−7.77 ± 1.80	−7.90 ± 2.22
3,4-Dihydroxybenzoic acid (**5**) (5 μM)	−28.28 ± 7.35	−18.37 ± 6.43
(−)-Bornesitol (**6**) (5 μM)	3.06 ± 0.45	0.89 ± 0.08
β-Sitostenone (7) (5 μM)	25.48 ± 6.30	35.86 ± 5.43
Kojic acid (10 μM)	25.38 ± 1.40	20.00 ± 2.12

Data in the table are means ± S.D. of *n* = 3; (−) means an increase in the tyrosinase activity or in increase the melanin content caused by the test compounds.

## 3. Experimental Section

### 3.1. General Procedures

UV (Ultraviolet, UV) spectra were obtained on a UV-240 spectrophotometer (Jasco, Easton, MD, USA) in MeCN. IR (Infrared, IR) spectra were measured on a Hitachi 260-30 spectrophotometer (Hitachi, Tokyo, Japan). ^1^H-NMR (Nuclear Magnetic Resonance, NMR) (400/500 MHz) and ^13^C-NMR (100 MHz), HSQC (Heteronuclear Single Quantum Correlation, HSQC), HMBC (Heteronuclear Multiple Bond Correlation, HMBC), COSY (Correlation Spectroscopy, COSY) and NOESY (Nuclear Overhauser Effect Spectroscopy, NOESY) spectra were obtained on a Varian (Unity Plus) NMR spectrometer (Varian, Palo Alto, CA, USA). For each sample, 128 scans were recorded with the following settings: 0.187 Hz/point; spectra width, 14,400 Hz; pulse width, 4.0 μs; relaxation delay, 2 s. Low-resolution ESI-MS (Electrospray ionization, ESI) spectra were obtained on an API 3000 (Applied Biosystems, Foster City, CA, USA) and high-resolution ESI-MS spectra on a Bruker Daltonics APEX II 30e spectrometer (Bruker, Bremen, Germany). Silica gel 60 (Merck, 70–230 mesh, 230–400 mesh) was used for column chromatography. Precoated silica gel plates (Merck, Kieselgel 60 F-254), 0.20 mm and 0.50 mm, were used for analytical Thin Layer Chromatography (TLC) and preparative TLC, respectively, and visualized with 10% H_2_SO_4_.

### 3.2. Plant Material 

The roots of *Michelia compressa var. lanyuensis* were collected from Chiayi County, Taiwan, in March 2008. Plant material was identified by Dr. Fu-Yuan Lu (Department of Forestry and Natural Resources College of Agriculture, National Chiayi University, Chiayi, Taiwan). A voucher specimen (*M. compressa var. lanyuensis*) was deposited in the School of Medical and Health Sciences, Fooyin University, Kaohsiung, Taiwan.

### 3.3. Extraction, Isolation and Identification

The air-dried roots of *M. compressa var. lanyuensis* (5.1 kg) were extracted with MeOH (30 L × 4) at room temperature and a MeOH extract (146.2 g) was thus obtained upon concentration under reduced pressure. The MeOH extract, suspended in H_2_O (1 L), was partitioned with CH_2_Cl_2_ (2 L × 5) to give fractions soluble in CH_2_Cl_2_ (84.8 g) and H_2_O. The CH_2_Cl_2_-soluble fraction was chromatographed over silica gel (500 g, 70–230 mesh) using *n*-hexane/CH_2_Cl_2_/MeOH mixtures as the eluent to produce ten fractions. Part of fraction 2 (6.3 g) was subjected to silica gel chromatography, by eluting with *n*-hexane-EtOAc (60:1), enriched gradually with EtOAc, to furnish five fractions (2-1−2-5). Fraction 2-2 (1.2 g) was further purified on a silica gel column using *n*-hexane/CH_2_Cl_2_ mixtures to obtain *p*-hydroxybenzaldehyde (**4**) (4 mg) and 3,4-dihydroxybenzoic acid (**5**) (5 mg). Fraction 2-4 (2.2 g) was further purified on a silica gel column using *n*-hexane/EtOAc mixtures to obtain β-sitostenone (**7**) (22 mg). Part of fraction 5 (7.3 g) was subjected to silica gel chromatography by eluting with *n*-hexane-EtOAc (50:1), enriched with EtOAc to furnish five further fractions (5-1−5-5). Fraction 5-1 (2.1 g) was further purified on a silica gel column using *n*-hexane/EtOAc mixtures to obtain liriodenine (**1**) (14 mg). Part of fraction 5-3 (1.7 g) was further purified on a silica gel column using *n*-hexane/EtOAc mixtures to obtain pressalanine A (**3**). Part of fraction 8 (6.1 g) was subjected to silica gel chromatography by eluting with *n*-hexane-EtOAc (20:1), enriched with EtOAc to furnish three further fractions (8-1−8-5). Fraction 8-2 (2.1 g) was further purified on a silica gel column using CH_2_Cl_2_/MeOH mixtures to obtain (−)-*N*-acetylanonaine (**2**) (8 mg). Part of fraction 10 (12.5 g) was subjected to silica gel chromatography by eluting with *n*-hexane-EtOAc (20:1), enriched with EtOAc to furnish seven further fractions (10-1−10-7). Fraction 10-6 (3.8 g) was purified by recrystallization to obtain (−)-bornesitol (**6**) (35 mg).

*Liriodenine* (**1**). Yellow needles (CH_2_Cl_2_); M.P.:280–282 °C; UV λ_max_: 224, 248, 265, 308 nm; IR ν_max_: 1625 (-NHC=O), 1022, 933 (-OCH_2_O-) cm^−1^; ^1^H-NMR (500 MHz, CDCl_3_):δ 6.36 (2H, *s*, -OCH_2_O-), 7.14 (1H, *s*, H-3), 7.56 (1H, *t*, *J *= 8.0 Hz, H-9), 7.72 (1H, *t*, *J *= 8.0 Hz, H-10), 7.74 (1H, *d*, *J *= 5.0 Hz, H-4), 8.55 (1H, *d*, *J *= 8.0 Hz, H-8), 8.59 (1H, *d*, *J *= 8.0 Hz, H-11), 8.86 (1H, *d*, *J *= 5.0 Hz, H-5); ESI-MS *m*/*z*: 275 [M]^+^.

*(−)-N-Acetylanonaine* (**2**). Colorless needles (CH_2_Cl_2_); M.P.: 236‒237 °C; ${\left[\alpha \right]}_{D}^{25}$: −175° (c 0.05, CH_2_Cl_2_); UV λ_max_: 236, 275, 320 nm; IR ν_max_: 1630 (-NHC=O), 1042, 930 (-OCH_2_O-) cm^−1^; ^1^H-NMR (500 MHz, CDCl_3_): δ 2.19 and 2.22 (3H, *s*, NCOCH_3_), 2.63 and 2.69 (1H, *d*, *J *= 15.4 Hz, H-4α), 2.82 and 3.18 (1H, *d *and *m*, *J *= 14.0 Hz, H-7β), 2.82 and 2.86 (1H, *m*, H-4β), 2.86 and 3.18 (1H, *m *and *dd*, *J *= 14.0, 4.0 Hz, H-7α), 3.12 and 3.30 (1H, *m *and *t*, *J *= 12.6 Hz, H-5β), 3.99 and 4.70 (1H, *d *and *d*, *J *= 13.5 Hz and 14.0 Hz, H-5α), 4.95 and 5.21 (1H, *d *and *dd*, *J *= 7.0 Hz and 13.5, 3.5 Hz, H-6a), 5.97 and 6.09 (2H, *s*, -OCH2O-), 6.58 and 6.62 (1H, *s*, H-3), 7.32 (3H, *m*, H-8, 9, 10), 8.11 (1H, *m*, H-11); ESI-MS *m*/*z*: 307 [M]^+^.

*Pressalanine A* (**3**). White syrup; UV λ_max_: 233, 253, 290, 330, 385 nm; IR ν_max_: 3400 (OH), 1670 (-NHC=O), 1065, 920 (-OCH_2_O-) cm^−1^; ^1^H-NMR (400 MHz, CDCl_3_): δ 6.26 (2H, *s*, -OCH_2_O-), 7.06 (1H, *s*, H-7), 7.58 (2H, *m*, H-9,10), 7.69 (1H, *m*, H-8), 9.03 (1H, *m*, H-11); ESI-MS *m*/*z*: 279 [M]^+^.

*p-Hydroxybenzaldehyde* (**4**). Brown powder; M.P.: 115‒117 °C; UV λ_max_: 221, 284 nm; IR ν_max_: 3200 (OH), 1670 (C=O) cm^−1^; ^1^H-NMR (500 MHz, CDCl_3_): δ 6.95 (2H, *d*, *J *= 8.5 Hz, H-3, 5), 7.80 (2H, *d*, *J *= 8.5 Hz, H-2, 6), 9.88 (1H, *s*, CHO); ESI-MS *m*/*z*: 122 [M]^+^.

*3,4-Dihydroxybenzoic acid *(**5**). Brown powder; M.P.:198‒200 °C; UV λ_max_: 220, 276, 315 nm; IR ν_max_: 3200 (OH), 1670 (C=O) cm^−1^; ^1^H-NMR (500 MHz, CDCl_3_): δ 6.92 (1H, *d*, *J *= 8.4 Hz, H-5), 7.41 (1H, *d*, *J *= 2.0 Hz, H-2), 7.60 (1H, *dd*, *J *= 8.4, 2.0 Hz, H-6); ESI-MS *m*/*z*: 154 [M]^+^.

*(−)-Bornesitol* (**6**). White needles (Pyridine); M.P.: 226‒228 °C; ${\left[\alpha \right]}_{D}^{25}$: −13° (c 0.12, water); IR ν_max_: 3300, 2900, 1500, 1350, 1100 cm^−1^; ^1^H-NMR (400 MHz, C_5_D_5_N): δ 3.92 (3H, *s*, C_1_-OCH_3_), 4.15 (1H, *t*, *J *= 9.2 Hz, H-1), 4.62 (1H, *t*, *J *= 9.2 Hz*,* H-2), 4.74 (1H, *m*, H-6), 4.75 (1H, *m*, H-3), 4.77 (1H, *m*, H-5), 4.79 (1H, *m*, H-4), 5.47 (5H, *br s*, OH); ESI-MS *m*/*z*: 194 [M]^+^.

*β*-*Sitostenone* (**7**). White needles (CH_2_Cl_2_); M.P.: 85‒86 °C; IR ν_max_: 1680, 1618, 1460, 1375 cm^−1^; ^1^H-NMR (400 MHz, CDCl_3_): δ 0.69 (3H, *s*, H-18), 0.82 (3H, *d*, *J* = 6.8, Hz, H-26), 0.84 (3H, *s*, H-27), 0.87 (3H, *t*, *J* = 7.0, Hz, H-29), 0.95 (3H, *d*, *J* = 5.6 Hz, H-21), 1.03 (3H, *s*, H-19), 5.73 (1H, *d*, *J* = 1.4 Hz, H-3); ESI-MS *m*/*z*: 412 [M]^+^.

### 3.4. Cell Culture

The B16F10 cell line was obtained from the American Type Culture Collection (ATCC, Manassas, VA, USA). The cells was cultured in complete Minimal Essential Eagle’s Medium (MEM) containing 10% Fetal Bovine Serum (FBS), 1.5 g/L NaHCO_3_,10 μg/mL penicillin, 10 μg/mL streptomycin, 2 mM l-glutamine and 0.25 μg/mL fungizone at 37 °C with 5% CO_2_ in a humidified incubator.

### 3.5. Cell Viability Assay—XTT Assay

The XTT (2,3-bis-(2-methoxy-4-nitro-5-sulfophenyl)-2*H*-tetrazolium-5-carboxanilide) assay was used to determine cell viability and proliferation. The cell lines were seeded in 96-well culture plates (1 × 104 cells/well). XTT were obtained from Sigma-Aldrich GmbH (Stenheim, Germany). After seeding cells for 24 h, various different concentrations of compounds were added. After the treatment, the medium was replaced with fresh medium without drugs. XTT reagent was added to each well and cultured for 3 h. The optical density (OD) values of the supernatant were measured at 492 nm and 690 nm (reference wavelength). All experiments were repeated at least three times.

### 3.6. Determination of Melanin Content

Briefly, we followed the previous method with minor modifications [14,15]. B16F10 cells (10^5^/well) were seeded into a 24 well culture dish with or without treatment in 300 μL of culture medium and incubated for 48 h. Cell pellets were dissolved in 2.0 N NaOH containing 10% dimethyl sulfoxide (DMSO) and heated at 80 °C for 1 h, and suspensions were clarified by centrifugation for 10 min at 10,000× *g*. Amounts of melanin in the NaOH solution were spectrophotometrically measured at 405 nm. A melanin standard curve was prepared by dissolving synthetic melanin in 0.01 M sodium carbonate (pH 7.8) and treated as above.

### 3.7. Tyrosinase Activity

The tyrosinase activity was estimated by measuring the rate of dopachrome formation, based on the method described previously with minor modifications [14]. Briefly, B16F10 cells (10^5^/well) were seeded into a 24 well culture dish with or without treatment in 300 μL of culture medium and incubated for 48 h. Cells were then solubilized in phosphate buffer (0.1 M; pH 6.8) containing 0.1% Triton X100. The enzyme extract of cellular lysate was added to 10 μL of 10 mm l-tyrosine and 10 mm l-dopa as substrates mixed in 0.1 m phosphate buffer (pH 6.8). This reaction was then incubated at 37 °C for 3 h in a dark environment, and the absorbance at 490 nm was measured on a spectrophotometer. Tyrosinase inhibitory activity was determined at 490 nm by the following equation: Tyrosinase inhibitory activity was calculated using the following formula:

Tyrosinase inhibition (%) = [1 − (OD_490_ nm of sample/OD_490_ nm of control)] × 100 (1)

### 3.8. Statistical Analysis 

All experiments were carried out at three times and at least triplicate. The results were expressed as the average of the mean values ± standard deviation (SD). Statistical differences were estimated by one-way analysis of variance (ANOVA) followed by Dunnett’s test. Analysis of the data was done with SigmaPlot software (Version 8.0, SPSS Scientific, Chicago, IL, USA) and SigmaStat (Version 2.03, SPSS Scientific) run on an IBM-compatible computer. Statistical significance is expressed as * p < 0.05.

## 4. Conclusions

This study reported the tyrosinase activity and melanin content in B16F10 cells of pure constituents isolated from *M. compressa var. lanyuensis*. In this study, we found that liriodenine (**1**), (−)-*N*-acetylanonaine (**2**), and β-sitostenone (**7**) inhibited tyrosinase activity and decreased melanin contents without cytotoxic properties. These results indicate the inhibitory effects of compounds from *M. compressa var. lanyuensis *on melanin synthesis. Hence, liriodenine (**1**), (−)-*N*-acetylanonaine (**2**), and β-sitostenone (**7**) could be applied as a type of dermatological anti-hyperpigmentation agent in skin care products.
